# Noise Sensitivities for an Atom Shuttled by a Moving Optical Lattice via Shortcuts to Adiabaticity

**DOI:** 10.3390/e22030262

**Published:** 2020-02-25

**Authors:** Xiao-Jing Lu, Andreas Ruschhaupt, Sofía Martínez-Garaot, Juan Gonzalo Muga

**Affiliations:** 1 School of Electric and Mechatronics Engineering, Xuchang University, Xuchang 461000, China; luxiaojing1013@163.com; 2 Department of Physics, University College Cork, T12 YN60 Cork, Ireland; aruschhaupt@ucc.ie; 3 Departamento de Química Física, UPV/EHU, Apdo 644, 48080 Bilbao, Spain; jg.muga@ehu.es

**Keywords:** shortcuts to adiabaticity, noise sensitivities, transport, optical lattice, invariant-based inverse engineering

## Abstract

We find the noise sensitivities (i.e., the quadratic terms of the energy with respect to the perturbation of the noise) of a particle shuttled by an optical lattice that moves according to a shortcut-to-adiabaticity transport protocol. Noises affecting different optical lattice parameters, trap depth, position, and lattice periodicity, are considered. We find generic expressions of the sensitivities for arbitrary noise spectra but focus on the white-noise limit as a basic reference, and on Ornstein–Uhlenbeck noise to account for the effect of non-zero correlation times.

## 1. Introduction

The current technical capabilities used to control the translational motion of optical-lattice potential traps for atoms make possible a plethora of applications in quantum science and technology. We shall focus on the use of the lattice as a conveyor belt to transport atoms, although lattices may as well be moved for other purposes, e.g., to study the stability of superfluidity [1] or, by periodic driving (shaking), to control different aspects of single atoms or many-body systems [2,3]. Optical lattices are interesting for transporting atoms because of several useful properties: The possibility to have hundreds or thousands of minima (even more within hollow fibers [4,5]), trapping forces that are much larger than in single beam optical tweezers, parameter flexibility including time-dependent control, or the possibility to implement lattices that depend on the internal state [6]. The atoms may be transported between a preparation area to a “science chamber” [4,7,8], and the coherent control of individual atoms has been demonstrated towards on-demand positioning and delivery and the design of quantum registers [9,10,11,12,13,14]. Other applications include guided interferometry and precision measurement [4,5,15,16], quantum computation schemes via messenger atoms among distant register qubits [17], quantum random walks [18,19], quantum simulators [20], catapulting (launching) atoms with specified velocities [10,21], the creation of entangled states [22,23], integrating cold atoms with photonic platforms [24], and implementing two-qubit quantum gates and gate arrays [22,25,26].

In most of the above applications fast transport processes are of interest, e.g., to achieve high computational speeds, to allow for many repetitions and improve signal-to-noise ratios, or to avoid decoherence, but only as long as high fidelities with respect to desired final states are achieved. Shortcuts to adiabaticity (STA) are a set of techniques devised to speed up slow adiabatic processes. They help to design fast and robust operations, see [27,28] for review. In particular, STA have been applied to design fast transport operations that leave the final state unexcited [29,30,31,32,33,34], or atom launching and stopping [32,35], see further references for abundant work on STA-mediated transport, in particular Table IV, and a list of STA-mediated transport experiments in Table V of [28].

Shortcuts provide ideal trajectories for the control parameters, but the results may be affected by noise and implementation imperfections that limit experimentally the coherence of the transport, visibilities, and fidelities. Ruschhaupt et al [36] introduced a “noise sensitivity” to quantify these effects as the second order term in the expansion of the final energy with respect to the perturbative noise, and demonstrated that the time dependence of the controls may be optimized to achieve robust protocols in operations on two-level systems, see also [37,38]. Lu and coworkers [39,40] studied the effect of spring-constant noise on STA-driven transport of trapped ions, distinguishing two types of contributions to the sensitivity: Static (independent of trap motion) and dynamical, with opposite behavior with respect to shuttling time. They also demonstrated that the excitation can be reduced by proper process timing and design of the trap trajectory.

In this work we shall find the sensitivities for STA-mediated transport of atoms in optical lattices with respect to noises in the three parameters of a moving optical lattice potential $A\sin^{2}\left(Kx+\Phi \right)$, namely, noises in the “amplitude” *A*, in the phase Φ, or in the wavenumber *K*, which affect, respectively, the trap depth, the trap position, and the lattice periodicity. Interestingly they have different effects and behaviors, in particular with respect to static and dynamical components. This information will be instrumental in identifying dominant sources of noise and mitigating their effects. To focus on the effect of these noises excluding other phenomena and to get analytical results with explicit dependences, we shall assume throughout the paper conditions such that a single atom is trapped in a given lattice site minimum, with negligible tunneling, interatomic interactions, and spontaneous emission. Internal-state dependence of the lattice is disregarded, in fact the internal state plays no role in the following and it is assumed to remain unchanged along the shuttling. Moreover a deep lattice is assumed, in a Lamb–Dicke regime where the relevant atomic motion is effectively governed by a harmonic trap. This last condition could be relaxed as explained in the final discussion.

In Section 2 we review for completeness the invariant-based inverse engineering of STA trap trajectories for a harmonic trap and the general form of the noise sensitivities for a transport protocol. In Section 3, we consider the three types of noise for *A*, *K*, and Φ. The noise spectrum may be arbitrary, but we pay special attention to the white noise limit and to Ornstein–Uhlenbeck noise as a simple generalization to account for the effect of colored noise with a non-zero correlation time.

## 2. Invariant-Based Inverse Engineering and Noise Sensitivities

### 2.1. Invariant-Based Inverse Engineering

Let us first review the basic dynamical equations for a particle of mass *m* trapped in a harmonic trap with angular frequency Ω(t) whose center moves along an arbitrary trajectory Q(t). Then we shall use this information to inverse engineer special trajectories $q_{0}\left(t\right)$ that shuttle the particle without final excitation [32]. Effective one-dimensional configurations are assumed throughout. The Hamiltonian in coordinate (*x*) representation is

(1) $$H_{0}\left(t\right)=\frac{p^{2}}{2m}+\frac{1}{2}m\Omega^{2}\left(t\right){\left[x-Q\left(t\right)\right]}^{2},$$

where *p* is the momentum operator. We may subtract the purely time-dependent term and use instead $H_{0}=H_{0}\left(t\right)-m\Omega^{2}\left(t\right)Q{\left(t\right)}^{2}/2$ to find the dynamics,

(2) $$H_{0}\left(t\right)=\frac{p^{2}}{2m}-F\left(t\right)x+\frac{m}{2}\Omega^{2}\left(t\right)x^{2}.$$

$F\left(t\right)=m\Omega^{2}\left(t\right)Q\left(t\right)$ is a homogeneous force throughout space.

This Hamiltonian has a quadratic Lewis–Riesenfeld invariant of the form [32,41,42,43]

(3) $$\begin{array}{cc} I(t) & =\frac{1}{2m}{\left\{\rho \left(t\right)\left[p-m{\dot{q}}_{c}\left(t\right)\right]-m\dot{\rho }\left(t\right)\left[x-{\dot{q}}_{c}\left(t\right)\right]\right\}}^{2}\\ & +\frac{1}{2}m\omega_{0}^{2}{\left[\frac{x-q_{c}\left(t\right)}{\rho (t)}\right]}^{2}, \end{array}$$

where $\omega_{0}$ is a constant, and “invariance” means that its expectation values remain constant for the states driven by $H_{0}$, i.e.,

(4) $$\frac{dI(t)}{dt}\equiv \frac{\partial I(t)}{\partial t}+\frac{1}{i\hbar }\left[I\left(t\right),H_{0}\left(t\right)\right]=0.$$

Assuming a quadratic-in-momentum ansatz for I(t) in this equation, it is found that ρ(t) and F(t) must satisfy the “Ermakov” and “Newton” equations

(5) $$\begin{array}{ccc} \ddot{\rho }\left(t\right)+\Omega^{2}\left(t\right)\rho & = & \frac{\omega_{0}^{2}}{\rho^{3}\left(t\right)},\\ {\ddot{q}}_{c}\left(t\right)+\Omega^{2}\left(t\right)q_{c}\left(t\right) & = & F(t)/m. \end{array}$$

Hereafter we conveniently choose $\omega_{0}=\Omega \left(0\right)$. ρ(t) is a scaling factor that determines the width of the eigenstates of the invariant and $q_{c}\left(t\right)$ is a classical trajectory for the forced oscillator, see Equation (5). The eigenstates of I(t), Equation (3), are centered at $q_{c}\left(t\right)$. The eigenvalues $\lambda_{n}$ of I(t) are constant, $I\left(t\right)\psi_{n}\left(t\right)=\lambda_{n}\psi_{n}\left(t\right)$, whereas the (orthogonal) eigenstates of the invariant, $\psi_{n}\left(t\right)$, are time dependent,

(6) $$\psi_{n}\left(x,t\right)=\frac{1}{\sqrt{\rho }}e^{\frac{im}{\hbar }\left[\frac{\dot{\rho }x^{2}}{2\rho }+\frac{({\dot{q}}_{c}\rho -\dot{\rho }q_{c})x}{\rho }\right]}\phi_{n}\left(\frac{x-q_{c}}{\rho }\right),$$

where $\phi_{n}\left(x\right)$ are the eigenstates of a static harmonic oscillator with angular frequency $\omega_{0}$. Arbitrary solutions of the time-dependent Schrödinger equation $i\hbar \partial_{t}\Psi \left(x,t\right)=H_{0}\left(t\right)\Psi \left(x,t\right)$ may be expanded using the “transport modes” $\Psi_{n}\left(x,t\right)\equiv e^{i\theta_{n}\left(t\right)}\psi_{n}\left(x,t\right)$, where the Lewis–Riesenfeld phases $\theta_{n}\left(t\right)$ are found so that each transport mode is itself a solution,

(7) $$\theta_{n}\left(t\right)=\frac{1}{\hbar }\int_{0}^{t}\langle \psi_{n}\left(t^{\prime }\right)|i\hbar \frac{\partial }{\partial t^{\prime }}-H_{0}\left(t^{\prime }\right)|\psi_{n}\left(t^{\prime }\right)\rangle dt^{\prime }.$$

Thus, $\Psi \left(x,t\right)=\sum_{n}c\left(n\right)e^{i\theta_{n}\left(t\right)}\psi_{n}\left(x,t\right),$ where the c(n) are time independent, and n=0,1,….

In a rigid harmonic trap we may simply set

(8) $$\Omega \left(t\right)=\omega_{0},\;\;\;\rho \left(t\right)=1.$$

To inverse engineer a trap trajectory $q_{0}\left(t\right)$ that would transport the particle without final excitations from $q_{0}\left(0\right)=0$ to $q_{0}\left(T\right)=d$ in a time *T*, we shall design first $q_{c}\left(t\right)$ and deduce $q_{0}\left(t\right)$ from the Newton Equation (5) with $F\left(t\right)=m\omega_{0}^{2}q_{0}\left(t\right)$. We impose the boundary conditions [32]

(9) $$\begin{array}{c} q_{0}\left(0\right)=q_{c}\left(0\right)=0,\;\;{\dot{q}}_{c}\left(0\right)=0,\\ q_{0}\left(T\right)=q_{c}\left(T\right)=d,\;\;{\dot{q}}_{c}\left(T\right)=0, \end{array}$$

so that I(t) and $H_{0}\left(t\right)$ commute at t=0 and t=T. Therefore the two operators share eigenvectors at those times and the initial eigenvectors evolve into final eigenvectors without excitation. (This can be seen in Equation (6) taking into account Equations (8) and (9).) Moreover, the continuity of $q_{0}\left(t\right)$ is guaranteed by the additional conditions

(10) $${\ddot{q}}_{c}\left(0\right)=0,\;\;{\ddot{q}}_{c}\left(T\right)=0.$$

Note that the boundary conditions (9) guarantee the absence of excitation so $q_{c}\left(t\right)$ can be interpolated among them with great freedom to produce many different and valid shortcuts.

### 2.2. Noise Sensitivity

Here we shall define noise sensitivities following [40] but for a more general scenario, namely, for a Hamiltonian (1) where both Ω(t) and Q(t) could be affected by classical noise around their noiseless values $\omega_{0}$ and $q_{0}\left(t\right)$. The origin of the noise in the harmonic model is that, as explained in the next section in detail, different parameters of the optical lattice potential may suffer from some noisy deviation from the ideal value. This deviation is represented by λξ(t), possibly multiplied by some appropriate dimensional factor depending on the parameter. λ is the dimensionless perturbative parameter that should be set to one at the end of the calculation, and ξ(t) is also dimensionless. ξ(t) is assumed to be unbiased, i.e., the average over noise realizations E[⋯] gives zero, and the (dimensionless) correlation function α is stationary,

(11) E[ξ(t)]=0,E[ξ(t)ξ(s)]=α(t−s).

We also assume that there is no noise at initial time, so the initial conditions for ρ(t) and $q_{c}\left(t\right)$ are fixed as

(12) $$\begin{array}{ccc} \rho (0) & = & 1,\;\dot{\rho }\left(0\right)=\ddot{\rho }\left(0\right)=0,\\ q_{c}\left(0\right) & = & 0,\;{\dot{q}}_{c}\left(0\right)={\ddot{q}}_{c}\left(0\right)=0. \end{array}$$

Now the auxiliary functions ρ(t) and $q_{c}\left(t\right)$ are expanded in powers of λ,

(13) $$\begin{array}{ccc} \rho (t) & = & \rho^{\left(0\right)}\left(t\right)+\lambda \rho^{\left(1\right)}\left(t\right)+\cdot \cdot \cdot ,\\ q_{c}\left(t\right) & = & q_{c}^{\left(0\right)}\left(t\right)+\lambda q_{c}^{\left(1\right)}\left(t\right)+\cdot \cdot \cdot . \end{array}$$

Assuming as well a series expansion of Ω(t) and Q(t) in λ, we get in zeroth order (noiseless limit)

(14) $$\begin{array}{ccc} \rho^{\left(0\right)}\left(t\right) & = & 1,\\ {\ddot{q}}_{c}^{\left(0\right)}\left(t\right)+\omega_{0}^{2}q_{c}^{\left(0\right)}\left(t\right) & = & \omega_{0}^{2}q_{0}\left(t\right), \end{array}$$

where $q_{c}^{\left(0\right)}\left(t\right)$ satisfies Eqautions (9) and (10).

We also assume that there is no noise at the final time, $H\left(T\right)=p^{2}/2m+m\omega_{0}^{2}{\left(x-d\right)}^{2}/2$. The expectation value of H(T) for a state $\Psi_{n}\left(T\right)=e^{i\theta_{n}\left(T\right)}\psi_{n}\left(T\right)$, see Equation (6), that started as the $n_{th}$ mode for a realization of the noise ξ(t) can be found exactly,

(15) $$\begin{array}{cc} E_{n,\xi } & =\langle H\left(T\right)\rangle =\langle \Psi_{n}\left(T\right)\left|H\left(T\right)\right|\Psi_{n}\left(T\right)\rangle \\ & =\frac{m}{2}\omega_{0}^{2}{\left[q_{c}\left(T\right)-d\right]}^{2}+\frac{\hbar \omega_{0}}{4}\left(2n+1\right)\frac{1+\rho^{4}\left(T\right)}{\rho^{2}\left(T\right)}\\ & +\frac{m}{2}{\dot{q}}_{c}^{2}\left(T\right)+\frac{\hbar }{4\omega_{0}}\left(2n+1\right){\dot{\rho }}^{2}\left(T\right). \end{array}$$

$E_{n,\xi }$ can be expanded in powers of λ as

(16) $$E_{n,\xi }\approx E_{n,\xi }^{\left(0\right)}+\lambda E_{n,\xi }^{\left(1\right)}+\lambda^{2}E_{n,\xi }^{\left(2\right)}+\cdot \cdot \cdot ,$$

with $E_{n,\xi }^{\left(1\right)}=\frac{\partial E_{n,\xi }}{\partial \lambda }$, $E_{n,\xi }^{\left(2\right)}=\frac{1}{2}\frac{\partial^{2}E_{n,\xi }}{\partial \lambda^{2}}$. Combining Equation (15) and the expansions for ρ(t) and $q_{c}\left(t\right)$ in Equation (13), we find the zeroth order $E_{n,\xi }^{\left(0\right)}=\hbar \omega_{0}\left(n+\frac{1}{2}\right)$ and $E_{n,\xi }^{\left(1\right)}=0$, as expected, as well as

(17) $$\begin{array}{cc} E_{n,\xi }^{\left(2\right)} & =\frac{1}{2}m\omega_{0}^{2}q_{c}^{\left(1\right)}{\left(T\right)}^{2}+\hbar \omega_{0}\left(2n+1\right)\rho^{\left(1\right)}{\left(T\right)}^{2}\\ & +\frac{1}{2}m{\dot{q}}_{c}^{\left(1\right)}{\left(T\right)}^{2}+\frac{\hbar {\dot{\rho }}^{\left(1\right)}{\left(T\right)}^{2}}{4\omega_{0}}\left(2n+1\right). \end{array}$$

Averaging over different realizations of the noise,

(18) $$E_{n}=E\left[E_{n,\xi }\right]=E_{n}^{\left(0\right)}+\lambda^{2}\frac{1}{2}E\left[\frac{\partial^{2}E_{n,\xi }}{\partial \lambda^{2}}\right],$$

where $E_{n}^{\left(0\right)}=E_{n,\xi }^{\left(0\right)}$.

The noise sensitivity for a given transport protocol is defined as the second order coefficient, so it has dimensions of energy,

(19) $$\begin{array}{cc} G(T;n) & =\frac{1}{2}E\left[\frac{\partial^{2}E_{n,\xi }}{\partial \lambda^{2}}\right]=E\left[E_{n,\xi }^{\left(2\right)}\right]\\ & =G_{1}+G_{2}. \end{array}$$

We have separated the contributions related to ρ and to $q_{c}$,

(20) $$\begin{array}{ccc} G_{1} & = & \hbar \left(2n+1\right)\left\{\omega_{0}E\left[\rho^{\left(1\right)}{\left(T\right)}^{2}\right]+\frac{1}{4\omega_{0}}E\left[{\dot{\rho }}^{\left(1\right)}{\left(T\right)}^{2}\right]\right\},\\ G_{2} & = & \frac{1}{2}m\omega_{0}^{2}E\left[q_{c}^{\left(1\right)}{\left(T\right)}^{2}\right]+\frac{1}{2}mE\left[{\dot{q}}_{c}^{\left(1\right)}{\left(T\right)}^{2}\right]. \end{array}$$

In the following, we will discuss three different kinds of noise in the moving optical lattice and find the exact expressions of the corresponding sensitivities. To achieve robust protocols, the experimental goal is to minimize the total sensitivity $G_{1}+G_{2}$.

## 3. Noise in a Moving Optical Lattice

Let us consider an effective potential of the form

(21) $$\begin{array}{c} V=A\sin^{2}\left[Kx+\Phi \left(t\right)\right] \end{array}$$

due to a laser standing wave. All three coefficients could be affected by noise around central values *a*, *k* and ϕ so it is useful to consider an auxiliary “noiseless version” of Equation (21),

(22) $$\begin{array}{c} V\left(\mathrm{noise}\;\mathrm{free}\right)=a\sin^{2}\left[kx+\phi \left(t\right)\right]. \end{array}$$

Among the periodic minima we pick up the one at Q(t)=−Φ(t)/K as the one “occupied” by an atom. Expanding around this point we find a quadratic approximation for Equation (21),

(23) $$\begin{array}{ccc} A\sin^{2}\left[Kx+\Phi \left(t\right)\right] & \approx & AK^{2}{\left[x-Q\left(t\right)\right]}^{2}, \end{array}$$

where *A* is the potential depth of the lattice and *K* is the wavenumber of the laser light. Considering the possible time dependences, noisy or otherwise, the quadratic Hamiltonian may be written as Equation (1) with $\frac{1}{2}m\Omega {\left(t\right)}^{2}=AK^{2}$. Without any noise $\Omega \left(t\right)=\omega_{0}$, $\frac{1}{2}m\omega_{0}^{2}=ak^{2}$, and $Q=q_{0}$.

### 3.1. Wavenumber (Accordion) Noise

**Figure 1:**
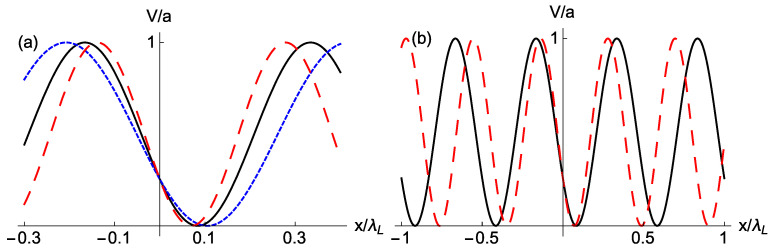
(Color online) Schematic effect of accordion (*K*) noise. Accordion noise consists of random compressions/expansions with respect to the pivot point x=0. (**a**) In a particular minimum, the one at $q_{0}>0$ without noise, expansions imply smaller trap frequencies together with displacements to the right, and compressions the opposite phenomena. The displacements of the minimum due to *K* noise increase with the distance to the pivot. The black solid line is the noiseless trap at some time during transport. The red dashed line represents a compression and the blue dotted line an expansion. The parameter values are chosen to easily visualize the effect and do not intend to be realistic. (**b**) Several lattice periods for the reference potencial without noise (black solid line) and the compressed version (red dashed line).

Accordion lattices have been implemented in different ways [44,45,46,47] to change the lattice periodicity keeping other parameters fixed. We consider first that the wave vector suffers from an involuntary “accordion noise” as K=k[1+λξ(t)], whereas A=a and Φ=ϕ. Some possible realizations of the potential at a given time are depicted in Figure 1 for a particular well (a) or for several wells (b). The harmonic potential with *K* noise now can be written as

(24) $$\begin{array}{cc} V & =ak^{2}{\left[1+\lambda \xi (t)\right]}^{2}{\left[x+\frac{1}{1+\lambda \xi (t)}\frac{\phi (t)}{k}\right]}^{2}\\ & =\frac{1}{2}m\omega_{0}^{2}{\left[1+\lambda \xi (t)\right]}^{2}{\left[x\;-\frac{q_{0}\left(t\right)}{1+\lambda \xi (t)}\right]}^{2}\\ & =\frac{1}{2}m\Omega^{2}\left(t\right){\left[x-Q\left(t\right)\right]}^{2}, \end{array}$$

where $\Omega^{2}\left(t\right)=\omega_{0}^{2}{\left[1+\lambda \xi \left(t\right)\right]}^{2}$, whereas the minimum at $Q\left(t\right)=q_{0}\left(t\right)/\left(1+\lambda \xi (t)\right)$ is displaced by the noise proportionally to $q_{0}\left(t\right)$. Both the spring constant and the trap position are affected by the accordion noise.

Substituting the expansions of ρ(t) and $q_{c}\left(t\right)$ of Equation (13) into Equation (5), and keeping only the first order in λ, $\rho^{\left(1\right)}\left(t\right)$ and $q_{c}^{\left(1\right)}\left(t\right)$ will satisfy

(25) $$\begin{array}{ccc} {\ddot{\rho }}^{\left(1\right)}\left(t\right)+4\omega_{0}^{2}\rho^{\left(1\right)}\left(t\right)\;\; & = & \;\;-2\omega_{0}^{2}\xi \left(t\right),\\ {\ddot{q}}_{c}^{\left(1\right)}\left(t\right)+\omega_{0}^{2}q_{c}^{\left(1\right)}\left(t\right)\;\; & = & \;\;[{\ddot{q}}_{c}^{\left(0\right)}\left(t\right)-\omega_{0}^{2}q_{c}^{\left(0\right)}\left(t\right)]\xi \left(t\right), \end{array}$$

with initial conditions $\rho^{\left(1\right)}\left(0\right)={\dot{\rho }}^{\left(1\right)}\left(0\right)={\ddot{\rho }}^{\left(1\right)}\left(0\right)$ and $q_{c}^{\left(1\right)}\left(0\right)={\dot{q}}_{c}^{\left(1\right)}\left(0\right)={\ddot{q}}_{c}^{\left(1\right)}\left(0\right)$. The solutions of Equation (25) are

(26) $$\begin{array}{ccc} \rho^{\left(1\right)}\left(t\right) & = & -\omega_{0}\int_{0}^{t}ds\;\xi \left(s\right)\sin\left[2\omega_{0}\left(t-s\right)\right],\\ q_{c}^{\left(1\right)}\left(t\right) & = & \frac{1}{\omega_{0}}\int_{0}^{t}ds\;\xi \left(s\right)\left[{\ddot{q}}_{c}^{\left(0\right)}\left(s\right)-\omega_{0}^{2}q_{c}^{\left(0\right)}\left(s\right)\right]\sin\left[\omega_{0}\left(t-s\right)\right]. \end{array}$$

Substituting them into Equation (20) and using Equation (11), we get the sensitivity

(27) $$\begin{array}{ccc} G(T;n)\;\; & = & \;\;G_{1K}\left(T;n\right)+G_{2K}\left(T;n\right),\\ G_{1K}\left(T\right)\;\; & = & \;\;\hbar \omega_{0}^{3}\left(4n+2\right)\int_{0}^{T}ds\;\alpha \left(s\right)\left(T-s\right)\cos\left(2\omega_{0}s\right),\\ G_{2K}\left(T\right)\;\; & = & \;\;m\;\int_{0}^{T}\;\;\;ds\;\alpha \left(s\right)f_{K}\left(s\right), \end{array}$$

where

(28) $$f_{K}\left(s\right)=\cos\left(\omega_{0}s\right)\int_{0}^{T-s}B\left(u\right)B\left(u+s\right)du,$$

with $B\left(u\right)={\ddot{q}}_{c}^{\left(0\right)}\left(u\right)-\omega_{0}^{2}q_{c}^{\left(0\right)}\left(u\right)$. $G_{1K}$ is independent of the trajectory, it is a “static” contribution that depends on *n*, the frequency $\omega_{0}$, the correlation function of the noise α(t), and shuttling time *T*. Instead, $G_{2K}$ is a “dynamical” contribution that depends on the trajectory, on α(t), and on the mass *m*. The static/dynamical character can be traced back to Equation (25). The noise forcing term in the equation for $\rho^{\left(1\right)}$ does not depend on the trajectory whereas the one for $q_{c}^{\left(1\right)}$ does. However $G_{1}$ and $G_{2}$ in Equation (20) do not necessarily become, respectively, static and dynamical sensitivities for all noises as they do here, see in particular Section 3.3 on “position noise” below. Each noise type requires a separate analysis.

To evaluate the integrals in Equation (27) the correlation function α(t) of the noise has to be specified. We consider Ornstein–Uhlenbeck (OU) noise with correlation function

(29) $$\alpha \left(t\right)=\frac{D}{2\tau }e^{-t/\tau }$$

as a simple, natural generalization of Gaussian white noise to introduce a finite correlation time τ. *D*, with dimensions of time, sets the strength of the noise (the factor *D* was taken out of the correlation function in [40] (when comparing the present work and [40] note also that λ had dimensions of square root of time there, whereas it is dimensionless here). The convention here is as in [39]). OU noise is not the most general colored noise, but it covers a much larger domain than the white-noise assumption [48]. When τ→0, it reduces to white noise, and is also instrumental in generating flicker noise by superposing a range of correlation times [39].

**Figure 2:**
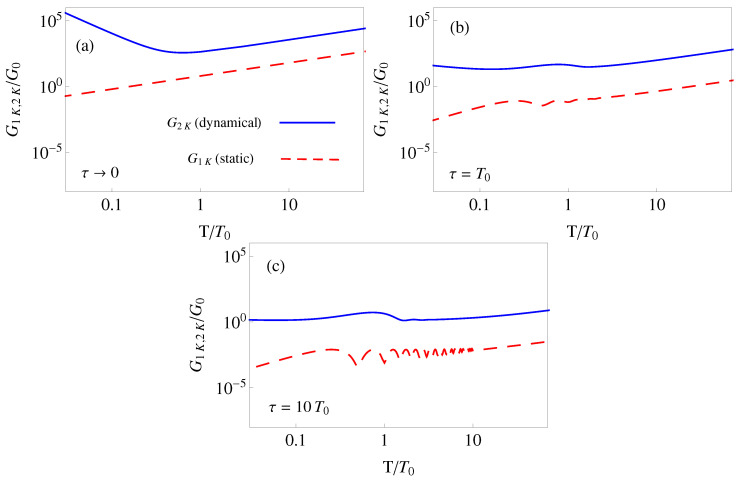
(Color online) Log–log plot of the sensitivity to accordion noise in units of $G_{0}=\hbar \omega_{0}^{2}D$ for a polynomial protocol versus final time in units of the oscillation period $T_{0}=2\pi /\omega_{0}$ and for different values of τ: (**a**) τ→0 (white noise limit); (**b**) $\tau =T_{0}$; (**c**) $\tau =10T_{0}$. The blue solid line is the dynamical component $G_{2K}$ and the red dashed line the static component $G_{1K}$. The parameters are $\lambda_{L}=2\pi /k=866$ nm, $d=\frac{1}{2}\lambda_{L}$, $a=850\;E_{R}$, mass of ${}^{133}$Cs, n=0, $\omega_{0}=\sqrt{2ak^{2}/m}=2\pi \times 116$ kHz, and recoil energy $E_{R}={\left(\hbar k\right)}^{2}/\left(2m\right)$. The same scale is kept in these three figures and in later figures for the other noises (Figures 4 and 6) to compare easily the different sensitivities.

To be more specific and see the behavior of the sensitivity, we assume a simple polynomial ansatz, $q_{c}^{\left(0\right)}\left(t\right)=\sum_{j=0}^{5}b_{j}t^{j}$, where the $b_{j}$ are fixed to satisfy the imposed boundary conditions. The optical lattice moves in our simulations from 0 to $d=\lambda_{L}/2$, where $\lambda_{L}$ is the wavelength of the light creating the optical lattice, so that *d* is the distance between two contiguous minima. In Figure 2, the sensitivity components $G_{1K}$ and $G_{2K}$ are shown versus final time for a Cs atom, see further details in the caption. The lattice parameters are realistic and taken from [49]. They correspond to a Lamb–Dicke regime, $\hbar \omega_{0}/E_{R}\approx 58$, where $E_{R}={\left(\hbar k\right)}^{2}/\left(2m\right)$ is the recoil energy.

In Figure 2 we include small *T* values below the period $T_{0}=2\pi /\omega_{0}$ for completeness, but note that the harmonic and single well approximations will fail in such a regime. For a simple estimate of minimal allowed shuttling times we may compare a lower bound for averaged potential energy during transport [32], with the potential depth *a*, i.e., $6md^{2}/\left(T^{4}\omega_{0}^{2}a\right)\gg 1$ should hold for the particle to stay in a minimum. Using $\omega_{0}=\sqrt{2ak^{2}/m}$ and d=π/k gives a minimal time scale $T\approx T_{0}/2$. Shorter times which would not be affected by the failure of the harmonic approximation may be implemented by applying a time-dependent homogeneous force compensating the inertial force, this is discussed briefly in the final section, see also [32].

In the white noise limit τ→0 Equation (27) gives

(30) $$\begin{array}{ccc} G_{1K} & = & \hbar \omega_{0}^{3}D(2n+1)T,\\ G_{2K} & = & md^{2}D\left(\frac{181}{924}\omega_{0}^{4}T+\frac{60}{7T^{3}}+\frac{10\omega_{0}^{2}}{7T}\right), \end{array}$$

which implies a minimum for the dynamical term $G_{2K}$ at T≈0.63 $T_{0}$ and a monotonous growth with process time *T* for the static part $G_{1K}$. For $T>T_{0}$ both terms grow linearly with time *T* as shown in the right part of Figure 2a. Comparing $G_{1K}$ and the linear part of $G_{2K}$ we find that for this noise $G_{2K}$ is always dominant in the Lamb–Dicke regime. In the white noise limit, with d=π/k,

(31) $$\frac{G_{2K}\left(\mathrm{linear}\;\mathrm{in}\;T\;\mathrm{term}\right)}{G_{1K}}=\frac{181}{924}\frac{m\omega_{0}d^{2}}{\hbar }\approx 0.96\frac{\hbar \omega_{0}}{E_{R}}.$$

The effect of a finite correlation time with a OU correlation function is explored numerically in Figure 2b,c: Increasing correlation times diminish the sensitivity in all time *T* regions and even suppress strongly the short-time *T* growth of sensitivity characteristic of the white noise limit. $G_{2K}$ stays dominant over $G_{1K}$ for all τ.

The previous results are also consistent with the known effects of spring-constant noise in static traps [50,51]. We assume now no transport ($q_{0}\left(t\right)=0$ and $q_{c}\left(t\right)=0$) and consider the static part $G_{1}$ alone. Using Equations (18), (27) and (29) and assuming that T≫τ, we then arrive at

(32) $$\frac{dE_{n}}{dT}=4\omega_{0}^{2}\pi E_{n}^{\left(0\right)}S_{K}\left(2\omega_{0}\right),$$

where $S_{K}\left(2\omega_{0}\right)$ is the spectral density for the fractional fluctuation in the wavenumber at the second harmonic of the trap (we have set λ=1),

(33) $$S_{K}\left(2\omega_{0}\right)=\frac{1}{\pi }\int_{0}^{\infty }\alpha \left(t\right)\cos\left(2\omega_{0}t\right)dt,$$

see also the corresponding discussion for amplitude noise in the following subsection.

### 3.2. Amplitude (Trap Depth) Noise

**Figure 3:**
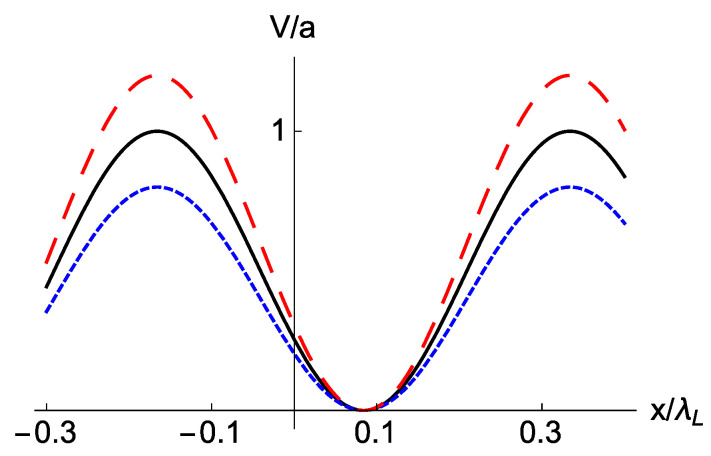
(Color online) Two realizations of the potential due to amplitude noise (red dashed line and dotted blue line) at some given time. The corresponding noiseless potential is also represented as a solid black line.

Trap depth noise may be due to laser intensity fluctuations as well as to pointing instabilities of the laser beams that could arise as a consequence of shifts of the laser beam, acoustic vibrations or air flow [11]. For example Kuhr et al. [11], estimated the fluctuations of the trap depth in their optical lattice setting to reach up to 3% for time scales t>100 ms. We consider amplitude noise as A=a[1+λξ(t)] (whereas K=k, and Φ=ϕ), see Figure 3, so that the optical lattice potential can be written as

(34) $$\begin{array}{c} V=a\left[1+\lambda \xi \left(t\right)\right]k^{2}{\left(x-q_{0}\right)}^{2}=\frac{1}{2}m\Omega^{2}\left(t\right){\left(x-q_{0}\right)}^{2}, \end{array}$$

where $\Omega^{2}\left(t\right)=\omega_{0}^{2}\left[1+\lambda \xi \left(t\right)\right]$ is affected by a classical spring constant noise.

Similarly to the procedure followed for accordion noise, substituting the expansions of ρ(t) and $q_{c}\left(t\right)$ into Equation (5), and keeping only the first order of λ, $\rho^{\left(1\right)}\left(t\right)$ and $q_{c}^{\left(1\right)}\left(t\right)$ will satisfy

(35) $$\begin{array}{ccc} {\ddot{\rho }}^{\left(1\right)}\left(t\right)+4\omega_{0}^{2}\rho^{\left(1\right)}\left(t\right) & = & -\omega_{0}^{2}\xi \left(t\right),\\ {\ddot{q}}_{c}^{\left(1\right)}\left(t\right)+\omega_{0}^{2}q_{c}^{\left(1\right)}\left(t\right) & = & {\ddot{q}}_{c}^{\left(0\right)}\left(t\right)\xi \left(t\right), \end{array}$$

with initial conditions $\rho^{\left(1\right)}\left(0\right)={\dot{\rho }}^{\left(1\right)}\left(0\right)={\ddot{\rho }}^{\left(1\right)}\left(0\right)$ and $q_{c}^{\left(1\right)}\left(0\right)={\dot{q}}_{c}^{\left(1\right)}\left(0\right)={\ddot{q}}_{c}^{\left(1\right)}\left(0\right)$. The solutions of Equation (35) are

(36) $$\begin{array}{ccc} \rho^{\left(1\right)}\left(t\right) & = & -\frac{\omega_{0}}{2}\int_{0}^{t}ds\;\xi \left(s\right)\sin\left[2\omega_{0}\left(t-s\right)\right],\\ q_{c}^{\left(1\right)}\left(t\right) & = & \frac{1}{\omega_{0}}\int_{0}^{t}ds\;\xi \left(s\right)\sin\left[\omega_{0}\left(t-s\right)\right]{\ddot{q}}_{c}^{\left(0\right)}\left(s\right). \end{array}$$

Substituting $\rho^{\left(1\right)}\left(t\right)$ and $q_{c}^{\left(1\right)}\left(t\right)$ into Equation (20), we get

(37) $$\begin{array}{ccc} G(T;n) & = & G_{1A}\left(T;n\right)+G_{2A}\left(T;n\right),\\ G_{1A}\left(T\right) & = & \hbar \omega_{0}^{3}\left(n+\frac{1}{2}\right)\;\;\int_{0}^{T}\;\;ds\;\alpha \left(s\right)\left(T\;-\;s\right)\cos\left(2\omega_{0}s\right),\\ G_{2A}\left(T\right) & = & m\;\int_{0}^{T}\;\;\;ds\;\alpha \left(s\right)f_{A}\left(s\right), \end{array}$$

where

(38) $$f_{A}\left(s\right)=\cos\left(\omega_{0}s\right)\;\int_{0}^{T-s}\;\;du\;{\ddot{q}}_{c}^{\left(0\right)}\left(u\right){\ddot{q}}_{c}^{\left(0\right)}\left(u+s\right).$$

As before we compute the integrals for OU noise, and use the polynomial ansatz for $q_{c}$. In the white noise limit τ→0

(39) $$\begin{array}{ccc} G_{1A} & = & \frac{D}{4}\hbar \omega_{0}^{3}\left(2n+1\right)T,\\ G_{2A} & = & \frac{D60md^{2}}{7T^{3}}. \end{array}$$

**Figure 4:**
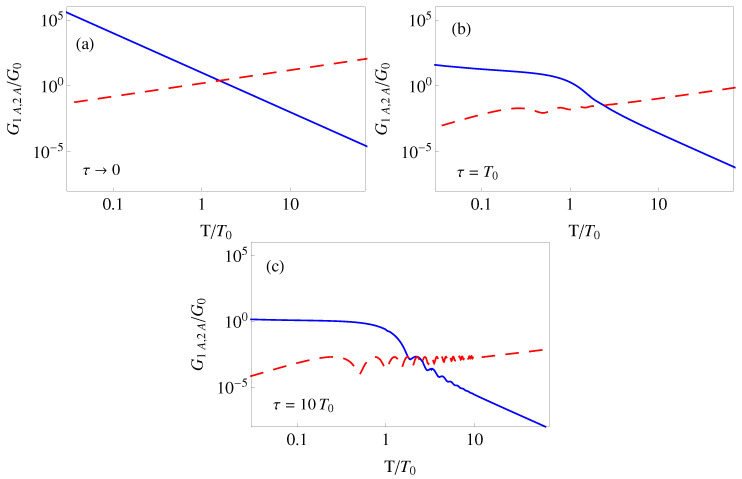
(Color online) Amplitude-noise sensitivity for a polynominal protocol versus final time (log–log plot) and for different correlation times τ: (**a**) τ→0; (**b**) $\tau =T_{0}$; (**c**) $\tau =10T_{0}$. Dashed red line: Static term $G_{1A}$; solid blue line: Dynamical term $G_{2A}$. The parameters and scales are the same as in Figure 2.

Up to the scaling due to the optical lattice parameters, these expressions coincide with the results given in [40] for “spring-constant noise”, and different limits and regimes were discussed there in detail. Here we note that different from the accordion noise sensitivities, $G_{1A}$ (static) and $G_{2A}$ (dynamical) behave in opposite ways to each other in all *T* domains, and cross at a special optimal time with minimal sensitivity, see Figure 4.

The static part alone (no transport, $q_{0}\left(t\right)=0$) implies for *T* larger than the correlation time a heating rate in agreement with [50,51],

(40) $$\frac{dE_{n}}{dT}=\omega_{0}^{2}\pi E_{n}^{\left(0\right)}S\left(2\omega_{0}\right),$$

where $S_{A}\left(2\omega_{0}\right)$ is the spectral density for the fractional fluctuation in the amplitude (trap depth) at the second harmonic of the trap,

(41) $$S_{A}\left(2\omega_{0}\right)=\frac{1}{\pi }\int_{0}^{\infty }\alpha \left(t\right)\cos\left(2\omega_{0}t\right)dt.$$

Equations (33) and (41) are in fact equivalent since both $S_{A}$ and $4S_{K}$ may be interpreted as the spectrum for the fractional fluctuation of the spring constant.

The effect of increasing τ using OU noise is again to diminish the sensitivities, and to suppress the growth of the dynamical sensitivity for small $T<T_{0}$, see Figure 4.

### 3.3. Phase (Trap Position) Noise

**Figure 5:**
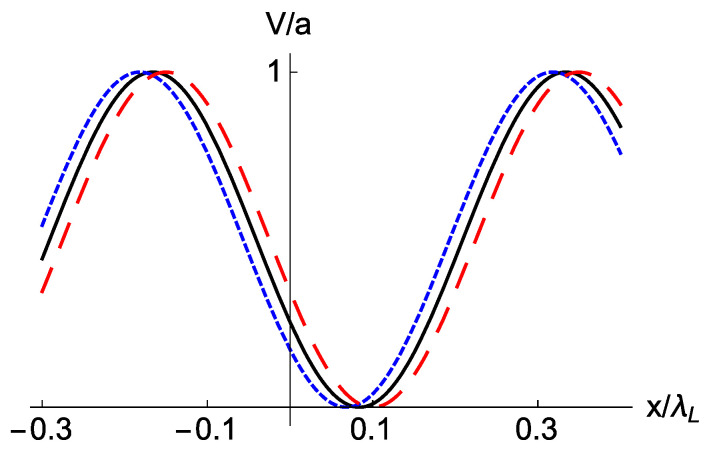
(Color online) Schematic representation of position noise in the optical lattice. The black solid line is the noiseless potential at some instant during the driving transport. The red dashed line and blue dotted line are two possible realizations of the potential due to position noise.

The standing wave phase ϕ(t) can be changed in time, moving the interference pattern, in different ways, e.g., [9,52]: One of the laser beams can be moved by mechanically moving a mirror [7]; the phase of one of the laser beams can be controlled with an electro-optical modulator; or a frequency mismatch Δν between the counterpropagating beams controlled by acousto-optical modulators produces a phase πΔνt. Of course all these methods are amenable to an imperfect control and fluctuations. Here we consider phase noise as Φ(t)=ϕ(t)−λξ(t) independent of other possible noises (A=a, K=k), see Figure 5. The harmonic potential takes now the form

(42) $$\begin{array}{c} \;\;V\;=\;ak^{2}\;{\left[x\;+\;\frac{\phi (t)\;-\;\lambda \xi (t)}{k}\right]}^{\;2}\;\;\;=\;\frac{m\omega_{0}^{2}}{2}{\left[x\;-\;q_{0}\left(t\right)\;-\;\frac{\lambda }{k}\xi \left(t\right)\right]}^{\;2}\;\;. \end{array}$$

The phase noise implies noise in the trap position, $Q\left(t\right)=q_{0}\left(t\right)+\frac{\lambda }{k}\xi \left(t\right)$.

First order equations are now

(43) $$\begin{array}{ccc} {\ddot{\rho }}^{\left(1\right)}\left(t\right)+4\omega_{0}^{2}\rho^{\left(1\right)}\left(t\right) & = & 0,\\ {\ddot{q}}_{c}^{\left(1\right)}\left(t\right)+\omega_{0}^{2}q_{c}^{\left(1\right)}\left(t\right) & = & \frac{\omega_{0}^{2}}{k}\xi \left(t\right), \end{array}$$

with initial conditions $\rho^{\left(1\right)}\left(0\right)={\dot{\rho }}^{\left(1\right)}\left(0\right)={\ddot{\rho }}^{\left(1\right)}\left(0\right)$ and $q_{c}^{\left(1\right)}\left(0\right)={\dot{q}}_{c}^{\left(1\right)}\left(0\right)={\ddot{q}}_{c}^{\left(1\right)}\left(0\right)$. The solutions of Equation (43) are

(44) $$\begin{array}{ccc} \rho^{\left(1\right)}\left(t\right) & = & 0,\\ q_{c}^{\left(1\right)}\left(t\right) & = & \frac{\omega_{0}}{k}\int_{0}^{t}ds\;\xi \left(s\right)\sin\left[\omega_{0}\left(t-s\right)\right], \end{array}$$

which give the sensitivities

(45) $$\begin{array}{ccc} G(T;n) & = & G_{1Q}\left(T;n\right)+G_{2Q}\left(T;n\right),\\ G_{1Q}\left(T\right) & = & 0,\\ G_{2Q}\left(T\right) & = & \frac{m\omega_{0}^{4}}{k^{2}}\int_{0}^{T}ds\;\alpha \left(s\right)\left(T-s\right)\cos\left(\omega_{0}s\right). \end{array}$$

The position noise sensitivity depends on the factor $m\omega_{0}^{4}/k^{2}$, α, and *T*. There is only a static contribution which, for this noise, depends on $G_{2}$ rather than on $G_{1}$ as in the previous two noises. Note also the independence on *n* of $G_{2Q}$ unlike the static terms $G_{1K}$ and $G_{1A}$. For a transport process the way to diminish its effect is to shorten the transport time.

**Figure 6:**
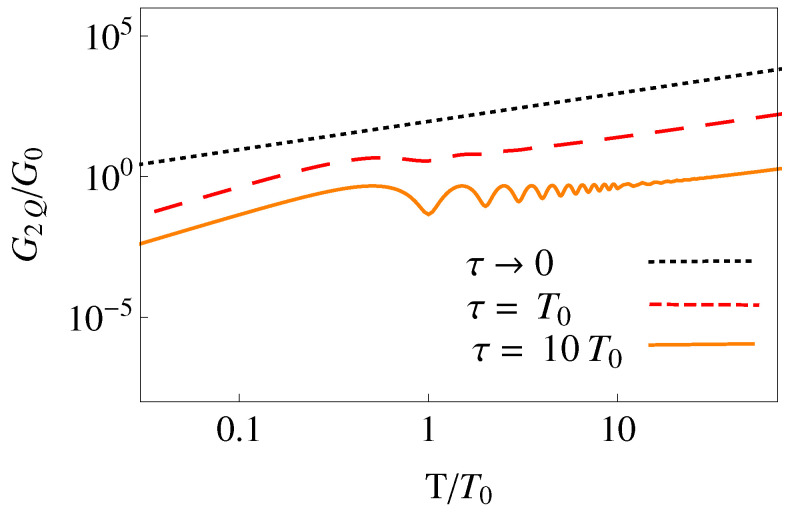
(Color online) Position-noise sensitivity for a polynomial transport protocol versus final time (log–log plot). The parameters and the scales are the same as in Figure 2.

As for the two previous noises we consider OU noise to compute the integral in Equation (45). In the white noise limit,

(46) $$G_{2Q}=\frac{m\omega_{0}^{4}}{2k^{2}}DT$$

as shown in Figure 6. Increasing τ diminishes the sensitivity and also affects the slopes differently for *T* larger or smaller than $T_{0}$.

For times *T* larger than the correlation time we find in second order, in agreement with [50,51], the heating rate

(47) $$\frac{dE_{n}}{dT}=m\omega_{0}^{4}\pi S_{Q}\left(\omega_{0}\right),$$

where $S_{Q}\left(\omega_{0}\right)$ is now the spectral density for the fluctuation of the trap position (we set λ=1, otherwise multiply by $\lambda^{2}$),

(48) $$S_{Q}\left(\omega_{0}\right)=\frac{1}{\pi }\int_{0}^{\infty }\frac{1}{k^{2}}\alpha \left(t\right)\cos\left(\omega_{0}t\right)dt.$$

## 4. Discussion

In this paper we have found the energy sensitivities with respect to noise in a conveyor-belt optical lattice that moves according to shortcut-to-adiabaticity protocols to transport atoms. The three types of noise considered affect the periodicity, the trap depth, or the trap position. A broad range of experimental settings may lead to these noises, to different combinations, or even to other noise forms (e.g., rocking). While the detailed analysis of the experimental settings is out of the scope of this work, the dependences found for the sensitivities will help to make a proper diagnosis of the predominant noise type and to implement mitigation strategies. Position noise is only affected by the static sensitivity which grows linearly with the shuttling time independently of the trajectory so the noise effect can only be mitigated by shortening the process time. Trap depth noise shows a more complex scenario for the sensitivity with a minimum at a specific shuttling time with dynamical effects dominating at very short times and static ones at long times. To locate the shuttling time where the sensitivity is minimal the analysis in [40] for spring constant noise is applicable. Dynamical sensitivities can in principle be diminished by optimizing the trajectory, a task left for future work. Accordion noise is dominated by the dynamical sensitivity at all shuttling times which also shows a minimum.

The existence of sensitivity minima demonstrates that the naive expectation that a smaller process time is always beneficial to combat the deleterious effects of noise is not necessarily true. Each type of noise requires a separate analysis and may or may not fulfill this expectation. It is interesting to compare the dominant sensitivities due to different noises in the regime $T>T_{0}$. In all cases they grow linearly with time for white noise. In the Lamb–Dicke regime the amplitude noise is found to have a weaker effect (although increased by *n*) than the other two, which behave similarly, see Equations (30), (39) and (46): $G_{2Q}/G_{1A}=\hbar \omega_{0}/\left[E_{R}\left(2n+1\right)\right]$, and $G_{2K}\approx 3.86\;G_{2Q}$.

A limitation of the shortcuts as implemented in Section 2.1 is that shuttling times shorter than an oscillation period break down the simplifying conditions assumed (motion in a single harmonic well). Shorter-time shortcuts may however be applied by compensating the inertial acceleration of the rigidly moving potential $U[q-q_{c}\left(t\right)]$ (the optical lattice potential) with an appropriate homogeneous force $-m{\ddot{q}}_{c}$ [31,32]. This trick does not require the trapping potential *U* to be harmonic, and wavefunctions that are initially stationary stay so during the whole transport in the frame moving with $q_{c}\left(t\right)$. In fact, the effective potential in the moving frame stays stationary, and “nothing happens” in that frame, apart from possible noises. Implementing this compensation may be technically challenging and to the best of our knowledge it has not been implemented yet for optical lattices, but the resulting benefits could make the effort worthwhile. We point out that there are different possibilities to implement the compensation, for example using lattice controlled rotations [45,46], or two optical lattices superposed with a large ratio of their periodicities, so that the one with the largest period provides an effectively linear potential.

Finally, the current noise analysis is also useful and applicable in the harmonic approximation to other transport platforms and systems such as atomic transport in moving magnetic microtraps in chips [53,54] or of ions in Paul traps [55,56,57].
